# Effects of the Inclusion of Ground *Pouteria sapota* Kernel on Intake, Digestibility, and Growth Performance in Lambs

**DOI:** 10.3390/ani12223154

**Published:** 2022-11-15

**Authors:** Adriana Sánchez-Zárate, Alfonso J. Chay-Canul, Edgar Aguilar-Urquizo, J. Roberto Sanginés-García, Víctor Manuel Moo-Huchin, Einar Vargas-Bello-Pérez, Ángel T. Piñeiro-Vázquez

**Affiliations:** 1 División de Estudios de Posgrado e Investigación, Tecnológico Nacional de México, Instituto Tecnológico de Conkal, Conkal 97345, Mexico; 2 División Académica de Ciencias Agropecuarias, Universidad Juárez Autónoma de Tabasco, Villahermosa 86040, Mexico; 3 Tecnológico Nacional de México, Instituto Tecnológico de Mérida, Mérida 97118, Mexico; 4 Department of Animal Sciences, School of Agriculture, Policy and Development, University of Reading, Earley Gate, P.O. Box 237, Reading RG6 6EU, UK

**Keywords:** by-product, kernel meal, growth performance, hair sheep, tropical regions

## Abstract

**Simple Summary:**

The concept of sustainable diets that are profitable, ethical, socioculturally acceptable, and environmentally beneficial is emerging as one of the key solutions to ensure the efficiency of livestock production systems. In this regard, agro-industrial by-products obtained from fruit processing have emerged as an alternative. Mamey pulp generates residual biomass from which the kernel is the main by-product that, due to its composition, can be used as ruminant feed. This study determined the effects of the inclusion of ground mamey kernel on intake, digestibility, and growth performance in lambs. No effects on nutrient intake or productive performance were observed. However, protein and fiber digestibility were reduced by ground mamey kernel inclusion. These findings suggest that mamey kernels can be included in ruminant diets.

**Abstract:**

This study determined the effect of replacing ground corn and soybean meal with ground *Pouteria sapota* kernel (PSSM) in lamb diets on nutrient intake and digestibility, performance, and carcass traits. Twenty-one male hair sheep lambs with an average body weight of 22 ± 3.5 kg were randomly assigned to three treatment diets containing PSSM at 0, 10, and 20% of the total dry matter (DM) inclusion. The study lasted 60 days, which included 15 days for adaption and 45 days for sample collection. The PSSM inclusion did not affect intake or performance (*p* > 0.05). However, ether extract (EE) digestibility linearly increased (*p* < 0.0001), while crude protein (CP) and acid detergent fiber (ADF) linearly decreased. Final body weight, total weight gain, average daily weight gain, feeding efficiency, and carcass traits were not affected by PSSM inclusion. In conclusion, these results suggest that PSSM can replace up to 200 g/kg DM of ground corn and soybean meal without affecting intake or animal performance.

## 1. Introduction

The constant growth of the human population, rising incomes, and urbanization have led to an increase in the demand for meat, milk, and eggs [1]. Nowadays, this increase in demand for animal source food has caused multiple challenges such as food insecurity (because animal feed rations contain products that can also serve as human food) and the environmental impact of land use and greenhouse gas emissions [2,3].

Feeding animals with food by-products and waste is a potential strategy that contributes to sustainable livestock production [4,5]. Compared with monogastric animals, the dietary supply of food by-products is practical for ruminants as their rumen contains a diverse microbial population that is able to metabolize nutrients and convert them into high-quality protein foods [6,7].

The use of agricultural by-products can improve an animal’s production efficiency and reduce food waste [8,9]. In ruminant diets, sources of protein and energy are based on expensive grains, cereals, and oilseeds [10,11]. Therefore, it is important to find alternative sources that can help to reduce the dependence on expensive imported feeds. In addition, current consumer trends show a demand for more sustainable and healthy foods [7,12].

Agro-industrial by-products have been used as feed alternatives for ruminants in countries where extreme climates cause pastures to be of lower nutritional quality and where cereals are expensive because they compete with human nutrition [13]. For ruminant diets, feedstuffs from fruit and vegetable processing plants have been successfully used [14,15,16,17]. Fruit processing results in several by-products such as peels, kernels, and unused biomass that can be used as livestock feed [18].

The mamey fruit (*Pouteria sapota*) is native to Mexico and Central America and is widely cultivated in tropical and subtropical regions [19]. Mexico produces more than 25,000 tons per year with the state of Yucatan as the main producer with more than 15 thousand tons per year [20]. Mamey is mostly consumed fresh or processed into jams and jellies. The pulp represents 65 to 70% of the fruit, while the kernel accounts for 3 to 8% [21]. The processing of mamey fruit pulp generates residual biomass and organic waste [22]. Therefore, the use of mamey by-products may result in a sustainable strategy that promotes the use of local resources and reduces pollution caused by the resulting organic waste from this industry.

In general, it is known that fruit kernels are rich in nutrients and a source of lipids, starch, protein, and many other compounds that can be used to feed ruminants without further processing [21]. Mamey kernel is a good source of fat (45%DM) and crude fiber (26%DM) [21]. This may lead to an improvement in growth rates in small ruminants as it can improve dietary energy density; however, no data are available related to animals fed with mamey kernel or its by-products. Oleic and linoleic acid are the most predominant fatty acids in mamey [23], and, during rumen biohydrogenation, these fatty acids can alter pathways that can lead to a reduction in meat saturated fatty acid contents [24]. High fat intakes can improve energy intake but may also lead to negative effects on nutrient digestibility [12]. Until now, the effects of dietary mamey kernel on nutrient digestibility in small ruminants remained unknown.

To the best of our knowledge, no investigations have been carried out to assess the effects of ground *Pouteria sapota* kernel (PSSM) on the diets of hair sheep lambs, which are animals resilient to high temperatures and the humidity of tropical regions. Compared with wool breeds, hair sheep are more prolific, rustic, and well-adapted to tropical conditions [25]. The objective of this study was to determine the effects of the dietary inclusion of PSSM on intake, digestibility, and growth performance in hair sheep lambs. In tropical regions of Mexico, ground corn is the main feedstuff used for feeding sheep, and PSSM appears to be a suitable alternative for replacing that dietary ingredient. Therefore, we hypothesized that replacing dietary ground corn with PSSM in hair sheep lamb diets would not compromise nutrient intake, digestibility, or productive performance.

## 2. Materials and Methods

### 2.1. Study Site

The experiment was carried out at the Instituto Tecnológico de Conkal (Merida, Mexico) (20°16′01.7″ N, 89°20′56.0″ W). The climate in the region is warm, with an average temperature of 26.8 °C and an average rainfall of 984.4 mm per year.

### 2.2. Ground Pouteria Sapota Kernel

**Figure 1:**
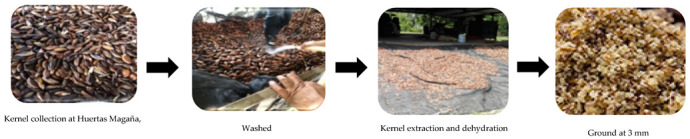
Processing of ground *Pouteria sapota* kernel.

The *Pouteria sapota* kernels used in this study were obtained from *Pouteria sapota* plantations of the Magaña I and Magaña II varieties. These were donated by the *Pouteria sapota* pulp processing plant of the Huertas Magaña (Akil, Mexico) company, located in Akil, Yucatán (20°16′01.7″ N, 89°20′56.0″ W), during the harvesting period of June 2021. The kernels were collected after processing the *Pouteria sapota* pulp and were later transferred to the facilities of the Tecnologico de Conkal. Here, the kernels were washed, manually extracted, and subjected to dehydration at 60 °C in an oven with forced air circulation. Finally, the kernels were ground to a particle size of 3 mm using a hammer mill (Figure 1).

### 2.3. Animals

Animals were handled according to the standards and procedures for handling experimental animals approved by the National Technological Institute of Mexico (Mexico City, Mexico) in the project “Productive behavior and meat quality of lambs fed with increasing levels of ground *Pouteria sapota* kernel” (ID 13289.21-P).

Twenty-one Pelibuey sheep with an average live weight of 22 ± 3.5 kg (mean ± standard deviation) were housed in individual cages in a roofed building with concrete floor. Before the study, animals were dewormed with Ivermectin^®^ (Laboratories Aranda, S.A. de C.V. México D.F., Ciudad de Mexico, Mexico) (1%; 1 mL for each 50 kg LW) and were injected with ADE vitamins (1 mL per each 10 kg LW). Pelibuey is a hair sheep breed native to Mexico that is found in tropical regions characterized by high temperature and humidity.

A completely randomized design with three treatments and seven replications per treatment was used [26]. The individual animal was considered the experimental unit. The animals were weighed at the beginning of the study and were assigned to each treatment, which consisted of different inclusion levels of PSSM (0, 10, and 20% of the DM). The study lasted 60 days, including a diet adaptation period of 15 days. Dietary ingredients and chemical composition are shown in Table 1.

### 2.4. Feed Intake

Feed offered and refusals were weighed every day. The nutrient intake and digestible dry matter intake were calculated based on the difference between the amount of each nutrient offered and orts (at least 10% DM offered previous day) [27].

### 2.5. Apparent Digestibility

Feces were collected daily at 8:00 and 16:00 during the last 7 days of the study. In the evening collection, both collections were weighed, and a 10% sample was taken and stored at −4 °C. Fecal samples were pooled for each treatment, and an aliquot of 10% was taken for chemical analyses to calculate apparent digestibility of each nutrient [28].

### 2.6. Daily Weight Gain and Feed Conversion Ratio

Animals were weighed every eight days prior to a 16 h fasting period. Daily weight gain (DWG) was determined by the difference between the final weight and the initial weight divided by the days elapsed in each weighing. Feed conversion ratio (FCR) was calculated based on feed intake and weekly live weight gain.

### 2.7. Water Intake

The amount of water consumed in a 24 h period was determined by the difference between offered and rejected water. Three containers with known amounts of water were placed as a reference, but without animal access, in which the loss by evaporation was measured, from which we could correct the amount consumed by the animals.

### 2.8. Carcass Characteristics

At the end of the feeding trial, lambs were slaughtered that same day in a commercial slaughterhouse in accordance with the Official Mexican Standards [29,30,31] established for the slaughtering and processing of animals for meat production.

The empty body weight (EBV) was calculated as the difference between the BW at slaughtering and the weight of the gastrointestinal tract (GT) content. The commercial carcass yield was calculated as hot carcass weight divided by the final live weight × 100. Then, carcasses were kept at 4 °C for 24 h for cold carcass weight recording and biological carcass yield calculation (cold carcass weight divided by the empty body × 100).

### 2.9. Chemical Analysis

Dry matter (DM) determination was carried out on forage samples in forced air over at 55 °C for 48 h (constant weight) (method no. 7.007 [32]). Nitrogen (crude protein = *N* × 6.25) concentration was carried out by combustion in a LECCO CN-2000 series 3740 (LECCO, Corporation) (method no. 2.057 [32]). Organic matter was determined by incineration in a muffle furnace at 550 °C for 6 h (method no, 923.03 [32]), and the contents of NDF and ADF were assayed as suggested by Van Soest et al. [33].

### 2.10. Statistical Analysis

The data were analyzed using the PROC MIXED procedure of SAS 9.4 ([26] SAS Institute, Inc., Cary, NC, USA). The data were analyzed according to the following model: Yi = µ + Ti + ei, where Yi is the observation, µ is the overall mean, Ti is the fixed effect of the inclusion of the *Pouteria sapota* kernel, and ei is the random error. The individual animal was considered the experimental unit. Treatment means were compared with the Tukey test with an alpha of 0.05; additionally, surface analyses were carried out to assess the linear, quadratic, or cubic trends of the response SAS 9.4 ([26] SAS Institute, Inc., Cary, NC, USA).

## 3. Results

### 3.1. Nutrient Intake

Compared with the control, the inclusion of PSSM increased ether extract contents (20.5 vs. 81.4 and 125.4 g/kg DM) (Table 1), and this was reflected in the increased ether extract intake from both diets containing ground *Pouteria sapota* kernel. The dietary inclusion of PSSM did not affect the intakes of DM, OM, CP, NDF, ADF, or water (*p* > 0.05) (Table 2).

### 3.2. Nutrient Digestibility

Regarding DM digestibility, a linear increase was observed (*p* < 0.05), while CP and ADF digestibility linearly decreased (*p* < 0.01). The NDF digestibility showed a quadratic effect as the PSSM content increased (*p* < 0.01) in the diets (Table 3).

### 3.3. Productive Performance and Carcass Characteristics

Initial body weight, final body weight, average daily gain, total weight gain, and feed conversion were similar between treatments (Table 4). Regarding carcass characteristics, the weight and yield of hot and cold carcasses were similar between treatments (*p* > 0.05) (Table 4).

## 4. Discussion

### 4.1. Nutrient Intake and Apparent Digestibility

The use of PSSM as a partial substitute for cereal grains used in the formulation of rations for ruminant diets represents a viable alternative because, in tropical regions, it is readily available. This by-product contains 15% crude protein (CP), 45% ether extract (EE), high concentrations of oil, and high contents of oleic and linoleic acids, where each gram of DM has an energy density of 5711 cal [23]. Overall, this is the first scientific report related to the effects of feeding PSSM to lambs. Our results support the study’s hypothesis, as PSSM could replace up to 200 g/kg DM of ground corn and soybean meal without affecting intake or animal performance.

The ground *Pouteria sapota* kernel used in this study had high levels of fat; however, the intakes of DM, OM, and CP were not affected by increasing levels of PSSM inclusion. However, a linear increase in EE intake was observed because the PSSM contained high contents of EE (45.2% of EE) [21]. The increasing intake of PSSM in hair sheep recorded in this study was somehow reflected in the increased DM and EE digestibility. However, it negatively affected the apparent digestibility of CP and ADF. Different results were observed by Lima et al. [34] when feeding sheep increasing levels of sunflower meal (0, 10, 20, and 30% of DM). They reported increases in the intake and digestibility of EE and a linear reduction in the digestibility of DM, which had a negative effect on weight gain and feed conversion. A possible cause of the reduced digestibility is the effect of PSSM polyunsaturated fatty acid contents on the membrane’s phospholipids of bacteria, which prevents growth and development of the microorganisms responsible for the degradation and fermentation of CP and ADF [35,36]. However, the lower digestibility of some components of the diet observed in this study did not affect the productive performance or carcass characteristics of hair sheep. It is important to carry out studies on inclusion levels higher than those used in this study to generate more information, as it is currently scarce on the use of PSSM as a partial substitute for oilseed grains and cereals. Although the inclusion of lipids in ruminant diets has been extensively studied and reviewed, our data on nutrient intake and apparent digestibility of lambs fed ground *Pouteria sapota* kernel provide new information on a feed matrix that may be considered “oily” or “greasy”. This information is extremely important in regions where mamey fruit and by-products are available.

### 4.2. Growth Performance

In this study, no negative effect was found on the growth performance between treatments. These results showed that although PSSM has large quantities of fat, at least under the conditions in this study, this did not negatively impact animal performance. A similar pattern was observed in weight gains, feed conversion, and carcass yields, which may have been influenced by the similar intakes of DM and CP between treatments. It should be noted that the inclusion of PSSM for each level represented 12 and 17% of EE, which did not affect lambs’ growth. It was reported that the inclusion of vegetable oil above 5% DM can affect ruminal fermentation due to the lipid coating of feed particles, thereby inhibiting bacterial enzymatic action [37]. Contrasting results have been reported in other studies that have included oilseeds. For example, Mach et al. [38] included flaxseed and canola seed at an inclusion level of 8% DM in steer diets and obtained daily weight gains and carcass yields similar to the control treatment. However, with an inclusion of 11% of the DM of flaxseed vegetable oil, Juarez et al. [39] reported decreases in weight gains and carcass yield. In another study carried out with palm oil by-products with a lipid inclusion between 7.4 and 10.9% in a diet for kids, lower values for daily weight gain (89 g/day) and a DM intake of 837 g/day were found [40]. These results were attributable to low nutrient digestibility and slow ruminal protein degradation. Likewise, in lambs, Lima et al. [34] observed negative effects on the productive parameters related to the decreased intake, due to the increased fiber and lipid contents associated with the reduced digestibility of dry matter and digestible carbohydrates when including 0, 10, 20, and 30% of sunflower seed cake (a maximum level of lipid inclusion of 6.4% in replacement for soybeans and corn). In general, the fact that ground *Pouteria sapota* kernel did not affect the lambs’ growth physiology was somewhat unexpected as it had 15% crude protein and 45% ether extract. Perhaps this was due to the number of animals used in the experiment.

The foregoing indicates that the effects of the inclusion of agro-industrial by-products as a source of energy through lipids in productive performance are complex and multifactorial. This is because these effects depend on the nature and quantity of lipids included as well as the characteristics and quantities of forages, concentrates, and minerals in the diet that influence the microbial ecosystem, the rate of passage, and the interaction of fatty acids with digestibility and ruminal fermentation kinetics [41]. This is the first study that reports the use of PSSM as an agro-industrial by-product in ruminant feed, and, according to our results, the inclusion of PSSM of up to 20% seems feasible for replacing ground corn and soybean meal without compromising feed intake or productive performance. This feeding strategy can help to mitigate the impact of feed costs, competition with food intended for human consumption, and the environmental impact of waste sources [7]. It is noteworthy to mention that PSSM is very low cost (0.15 USD /kg); in regions close to the study site, this represents a feasible alternative feedstuff to be included in lamb rations and to reduce production costs.

## 5. Conclusions

Overall, the results supported the study’s hypothesis, as ground *Pouteria sapota* kernel could replace up to 200 g/kg DM of ground corn and soybean meal without affecting intake or animal performance, but reduced the digestibility of CP and ADF. The inclusion of ground *Pouteria sapota* kernel increased intake and digestibility of ether extract and decreased the acid detergent fiber digestibility. However, it did not affect weight gain or carcass yield in hair sheep. In light of our findings, when available, ground *Pouteria sapota* kernel seems to be a feedstuff that can be used to increase energy density in hair sheep lamb diets instead of the conventional use of ground corn.

## Figures and Tables

**Table 1:** Ingredients and chemical compositions of experimental diets.

	Ground *Pouteria sapota* Kernel (%)		
	0	10	20
Ingredients (g/kg of DM)			
Straw	200	200	200
Sugar cane	100	100	100
Wheat bran	100	100	100
Ground corn	364	278	191
Ground *Pouteria sapota* kernel ^a^	0	100	200
Calcium carbonate	10	10	10
Soybean meal	205	198	191
Mineral and vitamin mix	21	21	21
Chemical composition (g/kg of DM)			
Dry matter (g/kg)	963	928	918
Organic matter	879	875	884
Crude protein	139	138	153
Ether extract	20.53	81.12	125.41
Neutral detergent fiber	246	211	230
Acid detergent fiber	142	124	119
Lignin	78.3	69.7	63.7

^a^ Containing 42% dry matter, 15% crude protein, 45% ether extract, 4% ash, 27% neutral detergent fiber, 13% acid detergent fiber, and 8% lignin.

**Table 2:** Effect of replacing ground corn and soybean meal with ground *Pouteria sapota* kernel (0, 10, and 20%) on nutrient intake in lambs.

	Ground *Pouteria sapota* Kernel (%)			SE	*p*-Value	Effects	
	0	10	20			Linear	Quadratic
Intake							
DM (kg/day)	1.53	1.49	1.40	0.11	0.725	0.45	0.86
DM (g/kg BW^0.75^)	117	111	106	7.48	0.651	0.36	0.99
OM (kg/day)	1.35	1.31	1.28	0.10	0.897	0.65	0.98
CP (kg/day)	0.22	0.21	0.22	0.01	0.913	0.80	0.75
EE (kg/day)	0.033	0.12	0.184	0.01	<0.0001	<0.0001	0.26
NDF (kg/day)	0.37	0.31	0.33	0.03	0.364	0.32	0.29
ADF (kg/day)	0.22	0.18	0.17	0.01	0.216	0.10	0.58
ME (MJ/day) *	16.37	15.56	15.35	1.33	0.857	0.60	0.85
	Water balance						
WI (L/day)	2.56	2.51	2.42	0.11	0.664	0.38	0.90
WI (mL/BW^0.75^)	195	189	186	8.07	0.713	0.43	0.83
WI (L/BW)	8.36	8.01	7.97	0.39	0.749	0.49	0.75
WI (L/kg DM intake)	1.73	1.74	1.79	0.11	0.904	0.71	0.82

SE, standard error; BW, body weight; BW^0.75^, metabolic body weight; DM, dry matter; OM, organic matter; CP, crude protein; NDF neutral detergent fiber; ADF, acid detergent fiber; EE, ether extract; WI, water intake. * Calculated as metabolic energy = 0.0157 × digestible organic matter (g/kg of DM).

**Table 3:** Effect of replacing ground corn and soybean meal with ground *Pouteria sapota* kernel (0, 10, and 20%) on digestibility coefficient of nutrients (g/kg) in lambs.

Items	Ground *Pouteria sapota* Kernel (%)			SE	*p*-Value	Effects	
	0	10	20			Linear	Quadratic
DM	739	747	753	4.77	0.140	0.05	0.77
OM	760	759	759	4.54	0.996	0.94	0.96
CP	977	724	759	4.48	0.0002	<0.0001	<0.0001
EE	864	959	964	13.3	<0.0001	<0.0001	<0.0001
NDF	391	296	396	12.3	<0.0001	0.80	<0.0001
ADF	312	186	173	14.9	<0.0001	<0.0001	0.006
DMDI	1130	1117	1055	89.3	0.811	0.55	0.84
OMDI	1030	997	971	82.3	0.886	0.63	0.97
CPDI	210	151	167	14.8	0.686	0.06	0.05
EEDI	26	118	177	0.90	<0.0001	<0.0001	0.15
NFDDI	148	91.4	129	12.8	0.019	0.30	0.01
ADFDI	68.3	32.8	30	6.26	0.0008	0.0005	0.046

SE, standard error; DM, dry matter; OM, organic matter; CP, crude protein; NDF neutral detergent fiber; ADF, acid detergent fiber; EE, ether extract; DMDI, dry matter intake digestibility; OMDI, organic matter digestibility intake; CPDI, EEDI, extract ether digestibility intake; NDFDI, neutral digestibility intake; ADFDI, acid detergent fiber intake.

**Table 4:** Effect of replacing ground corn and soybean meal with ground *Pouteria sapota* kernel (0, 10, and 20%) on productive performance and carcass characteristics of lambs.

	Ground *Pouteria sapota* Kernel (%)			SE	*p*-Value	Effects	
	0	10	20			Linear	Quadratic
Initial body weight (kg)	22.2	22.4	22.5	1.47	0.995	0.92	0.98
Final body weight (kg)	39.3	39.5	38.4	1.37	0.819	0.63	0.71
Average daily gain (g/day)	280	280	270	0.01	0.850	0.77	0.64
Total weight gain (kg)	17.1	17.0	15.9	0.88	0.558	0.36	0.60
Feed conversion	6.76	6.05	6.12	0.58	0.655	0.45	0.59
Carcass characteristics							
Hot carcass weight (kg)	21.1	18.7	19.6	1.11	0.346	0.36	0.24
Cold carcass weight (kg)	19.7	18.8	18.4	0.61	0.333	0.15	0.76
Hot carcass yield (%)	52.5	47.2	50.5	2.48	0.348	0.60	0.17
Cold carcass yield (%)	49.0	47.2	47.37	0.68	0.175	0.11	0.28

## Data Availability

Data will be available upon request to the corresponding authors.
